# Paper mulberry in animal nutrition: processing, host responses, and product quality

**DOI:** 10.3389/fvets.2026.1907163

**Published:** 2026-07-29

**Authors:** Ming Ma, Di Zhou, Rong Yang, Xuanxun Li, Xiangcai Chang, Chao Ban, Pipat Lounglawan

**Affiliations:** 1 College of Agriculture, Anshun University, Anshun, China; 2 Testing Center for Livestock and Poultry Germplasm, Guiyang, China; 3 Bijie Agricultural and Rural Affairs Service Center, Bijie, China; 4 School of Animal Technology and Innovation, Institute of Agricultural Technology, Suranaree University of Technology, Nakhon Ratchasima, Thailand

**Keywords:** *Broussonetia papyrifera*, functional feed, gut microbiota, meat quality, paper mulberry, silage

## Abstract

Paper mulberry (*Broussonetia papyrifera*) has attracted increasing attention as a local feed resource because it combines perennial woody biomass, protein-rich foliage and diverse plant secondary compounds. Its feeding value depends on plant part, harvest stage, processing method, fermentation quality, target animal species and dietary inclusion level. This review examines paper mulberry from feed composition and processing to animal responses, product traits and proposed mechanisms. Current evidence is strongest in ruminants, where paper mulberry silage or fermented feed has been associated with partial roughage replacement, maintained production and changes in rumen fermentation, antioxidant status, nitrogen utilization, milk fatty acids or meat quality under defined dietary conditions. Evidence in pigs, poultry, geese, fish and rabbits is more variable and suggests lower inclusion levels and stricter control of fibre, tannins and processing quality. Mechanistically, silage microbiota, tannin–protein interactions and rumen or hindgut microbial shifts are directly relevant to paper mulberry feeding, whereas nuclear factor erythroid 2-related factor 2/Kelch-like ECH-associated protein 1 (Nrf2/Keap1), nuclear factor kappa-B/mitogen-activated protein kinase (NF-κB/MAPK) and AMP-activated protein kinase–peroxisome proliferator-activated receptor–sterol regulatory element-binding protein 1 (AMPK–PPAR–SREBP1) pathways should be regarded as candidate explanations unless validated in target tissues. The main gaps include raw-material standardisation, fermentation-quality criteria, dose–response data, shelf-life endpoints and causal validation of proposed mechanisms.

## Introduction

Animal production is moving from expansion in output towards systems that also prioritise resource efficiency, product quality and lower environmental burden. Feed cost and feed security remain among the strongest constraints on this transition (1). Diets based on maize, soybean meal, alfalfa and maize silage also tie animal production to land use, trade volatility and competition for ingredients used in human food systems (2). The food-not-feed strategy argues for greater use of non-food-competing resources to reduce competition between humans and livestock, while work on ration diversity indicates that feed diversification can reduce system-level risk even when it increases formulation complexity (3, 4).

Woody forages have re-entered this discussion because they are perennial, regenerative, suited to marginal land, seasonally productive and able to fill local roughage gaps (5). Paper mulberry is one of the most prominent examples. In this review, “paper mulberry” is used as a general term for *Broussonetia papyrifera* and related hybrid paper mulberry materials unless a cited study specifically distinguishes hybrid material. Its chromosome-scale genome has provided biological explanations for traits linked to fibre formation, fast growth and adaptation, supporting its dual use as a forage and papermaking species (6). As a feed material, it brings together protein, structural carbohydrate, minerals and compounds such as flavonoids, polyphenols and tannins (6, 7). It is therefore better viewed as a nutrition-function composite resource rather than as a simple forage substitute.

A persistent ambiguity in this field is that paper mulberry is often described as a feed ingredient capable of replacing a given roughage or protein source, whereas animal responses vary substantially across trials (8, 9). This variability is not a binary question of efficacy. It reflects the strong dependence of paper mulberry value on processing and dose (9, 10). Leaves, petioles, stems and whole plants differ in nutrient composition (11, 12). Fresh, dried, ensiled and fermented products differ in palatability, protein degradation and microbial risk. Ruminants and monogastric species differ in their tolerance of fibre and tannins (13, 14). Paper mulberry is therefore better positioned as a processing-dependent functional woody feed resource, not as a single ingredient that can replace conventional feeds without constraint.

The scientific value of paper mulberry lies less in showing that animals can consume it than in defining the continuum linking raw-material heterogeneity, ensiling fermentation, gastrointestinal ecology, host metabolism and animal-product quality. Evidence from feed-resource science, silage fermentation, ruminant nutrition, pig and poultry production, meat, milk and egg quality, and cellular signaling argues for a shift from isolated substitution trials to integrated raw-material-processing-animal-product evaluation (11, 15–18).

## Literature search, study selection, and evidence appraisal

This review was designed as a structured narrative review. Literature was searched in PubMed, Web of Science, Google Scholar, CrossRef and China National Knowledge Infrastructure (CNKI) for studies published from 1 January 2010 to 1 June 2026. Studies were considered when full text was accessible and key information could be extracted. The search was limited to English and Chinese publications because these were the languages in which bibliographic information, full texts and methodological details could be reliably verified.

Search terms were combined using Boolean operators as follows: (“*Broussonetia papyrifera*” or “paper mulberry”) and (“animal nutrition” or “feed” or “silage” or “fermentation” or “ruminant” or “cattle” or “dairy cow” or “goat” or “sheep” or “pig” or “poultry” or “broiler” or “laying hen” or “goose” or “rabbit” or “fish” or “aquaculture”) and (“growth performance” or digestibility or “gut microbiota” or “antioxidant” or “inflammation” or “meat quality” or “milk quality” or “egg quality” or “fatty acid” or “shelf life”). Studies were included when they examined paper mulberry as a feed ingredient, silage, fermented feed, forage resource or biologically active feed material in animal production or feed-processing contexts. Studies were excluded when they focused only on human medicine, pure plant physiology, unrelated food chemistry, *in vitro* antioxidant activity without animal-feeding relevance, or duplicated data. Study selection proceeded by screening titles and abstracts, removing obviously irrelevant records, reading the full text of potentially eligible studies and retaining studies from which species, material form, inclusion level, processing method and at least one relevant endpoint could be extracted. A summary of the search strategy, selection logic and evidence-appraisal framework is provided in Supplementary Table S1 and Supplementary Figure S1.

The included literature was grouped into four levels according to evidential relevance. Level 1 comprised *in vivo* animal studies that reported the inclusion level, replacement target, animal species and at least one production, health or product-quality endpoint. Level 2 comprised studies of raw material, ensiling or fermentation, used to define processing quality, material heterogeneity and formulation boundaries. Level 3 comprised studies of product quality and host physiology, used to interpret the general biological processes shaping meat, milk and egg quality. Level 4 comprised phytochemistry, *in vitro* antioxidant work and canonical cell-signaling studies, which were used only to propose candidate mechanisms and not to support production claims on their own.

This four-level hierarchy was based on the proximity of each type of evidence to animal-feeding conclusions. *In vivo* feeding studies were placed at the highest level because they directly evaluate animal performance, physiological responses or product-quality traits. Feed-resource and fermentation studies were used to define the quality and processing background of the material, whereas host-physiology, product-quality, phytochemical and *in vitro* mechanistic studies were used mainly to support interpretation or generate hypotheses. This framework was intended to separate direct animal evidence from indirect mechanistic plausibility and to avoid overinterpreting cellular or *in vitro* findings as proven feeding effects. However, it is not a formal GRADE assessment, risk-of-bias evaluation or quantitative meta-analysis; therefore, it should not be used to generate pooled effect sizes or universal feeding recommendations, but rather to support evidence-informed interpretation and identify species-, processing- and dose-specific boundaries.

## Feed-resource attributes of paper mulberry

The first value of paper mulberry is its protein and amino-acid base. Across studies (Table 1), leaves generally contain more crude protein and less fibre than stems, while whole-plant material falls between these two fractions. Leaf crude protein is approximately 19.50–24.12% of dry matter, compared with only 5.67–8.49% in stems and 16.47–29.90% in whole plants. Similarly, leaf neutral detergent fibre is often 25.62–44.71% of dry matter, whereas stems may reach 58.87–68.06% (11, 12). Measurements of metabolizable energy in geese further show that even materials labelled as paper mulberry can differ in energy use according to leaf-stem ratio and origin (19).

**Table 1:** Nutrient composition of paper mulberry leaves, stems, and whole plants.

Reference	Plant part	DM (g/kg FM)	% DM						Key interpretation
			CP	EE	NDF	ADF	WSC	Ash	
Hao et al. (11)	Leaf	26.11	24.12	2.92	44.71	23.90	4.58	11.66	Protein-rich fraction; generally lower fibre than stems.
Han et al. (66)	Leaf	—	22.81	4.36	40.87	19.94	—	10.89	Protein-rich fraction; generally lower fibre than stems.
Osman et al. (67)	Leaf	—	19.50	6.55	43.20	34.15	—	13.86	Protein-rich fraction; generally lower fibre than stems.
Liu et al. (68)	Leaf	—	21.32	4.41	25.62	17.07	—	15.25	Protein-rich fraction; generally lower fibre than stems.
Wang et al. (69)	Leaf	16.18	23.62	—	31.65	20.15	5.20	—	Protein-rich fraction; generally lower fibre than stems.
Hao et al. (11)	Stem	27.11	8.49	2.28	68.06	56.59	1.16	4.73	High-fibre, low-protein fraction; limits high inclusion.
Liu et al. (68)	Stem	—	5.67	1.54	58.87	43.82	—	5.79	High-fibre, low-protein fraction; limits high inclusion.
Han et al. (66)	Stem	—	6.93	1.59	67.41	51.54	—	6.55	High-fibre, low-protein fraction; limits high inclusion.
Jiang et al. (12)	Stem	—	6.41	—	66.41	50.59	—	—	High-fibre, low-protein fraction; limits high inclusion.
Hao et al. (70)	Whole plant	30.18	19.72	—	38.92	20.46	6.29	—	Intermediate or variable composition reflecting leaf-to-stem ratio.
Li et al. (10)	Whole plant	21.60	23.50	—	33.10	19.60	7.49	—	Whole-plant material with relatively high CP; composition remains source-dependent.
Dong et al. (71)	Whole plant	22.53	29.41	—	42.94	14.81	7.98	—	Whole-plant material with relatively high CP; composition remains source-dependent.
Du et al. (15)	Whole plant	20.00	29.90	4.10	39.20	19.80	8.40	—	Whole-plant material with relatively high CP; composition remains source-dependent.
Hao et al. (11)	Whole plant	26.60	16.94	2.25	50.28	34.89	3.32	8.50	Intermediate or variable composition reflecting leaf-to-stem ratio.
Liu et al. (68)	Whole plant	—	17.38	3.09	34.24	23.77	—	13.07	Intermediate or variable composition reflecting leaf-to-stem ratio.
Han et al. (66)	Whole plant	—	17.07	3.42	45.63	26.94	—	10.38	Intermediate or variable composition reflecting leaf-to-stem ratio.
Si et al. (72)	Whole plant	21.18	18.55	3.05	44.41	22.14	4.18	11.67	Intermediate or variable composition reflecting leaf-to-stem ratio.
Sun et al. (29)	Whole plant	38.48	16.47	—	49.01	24.07	2.06	8.02	Intermediate or variable composition reflecting leaf-to-stem ratio.

ADF, acid detergent fiber; CP, crude protein; DM, dry matter; EE, ether extract; FM, fresh matter; NDF, neutral detergent fiber; WSC, water-soluble carbohydrate.

This heterogeneity means that paper mulberry cannot be formulated using a single average value. As shown in Table 2, leaves contain approximately 179.8–242.2 g/kg total amino acids, 84.8–105.2 g/kg essential amino acids and an essential-to-total amino-acid ratio of 43–47%. By contrast, stems contain only about 55.3 g/kg total amino acids, highlighting the influence of leaf proportion on protein quality (11, 12). Cutting height also modifies yield, nutrient composition, silage quality and *in vitro* fermentation, reinforcing the need to classify paper mulberry by plant fraction, harvest stage and processing method rather than by ingredient name alone (20).

**Table 2:** Amino acid composition of paper mulberry leaves, stems, and whole plants.

Reference	Plant part	g/kg		EAA/TAA	Notable amino-acid features	Key interpretation
		TAA	EAA			
Hao et al. (11)	Leaf	208.78	91.12	43.64%	Leucine, lysine, valine and threonine contribute substantially to EAA supply.	Higher total and essential amino acids.
Feng et al. (73)	Leaf	242.20	105.20	43.44%	Leucine, lysine, valine and threonine contribute substantially to EAA supply.	Higher total and essential amino acids.
Tu et al. (74)	Leaf	179.80	84.80	47.16%	Leucine, lysine, valine and threonine contribute substantially to EAA supply.	Higher total and essential amino acids.
Hao et al. (11)	Stem	55.32	22.05	39.86%	Low absolute amino-acid concentration; stem fraction is not a major protein source.	Low amino-acid density.
Hao et al. (11)	Whole plant	142.12	61.26	43.10%	Moderate amino-acid concentration; values depend on leaf-to-stem ratio and source.	Intermediate amino-acid profile.
Wang et al. (75)	Whole plant	120.30	53.40	44.39%	Moderate amino-acid concentration; values depend on leaf-to-stem ratio and source.	Intermediate amino-acid profile.
Wang et al. (75)	Whole plant	133.30	59.50	44.64%	Moderate amino-acid concentration; values depend on leaf-to-stem ratio and source.	Intermediate amino-acid profile.
Wang et al. (75)	Whole plant	122.80	54.20	44.14%	Moderate amino-acid concentration; values depend on leaf-to-stem ratio and source.	Intermediate amino-acid profile.
Wang et al. (75)	Whole plant	110.80	50.20	45.31%	Moderate amino-acid concentration; values depend on leaf-to-stem ratio and source.	Intermediate amino-acid profile.

EAA, essential amino acids; TAA, total amino acids. EAA was calculated as the sum of tryptophan, methionine, threonine, valine, isoleucine, leucine, phenylalanine, histidine, and lysine.

A second value lies in its bioactive chemistry. Phytochemical work on *Broussonetia* has reported flavonoids, flavonoid glycosides, lignans, phenolic acids, polysaccharides and condensed tannins, while several phenolic compounds isolated from leaves show *in vitro* antioxidant activity (21, 22). Antioxidant-guided chemical profiling has also shown that extracts from *Broussonetia* and Morus leaves share comparable antioxidant-active chemical bases (23). Such studies do not prove *in vivo* efficacy, but they provide substrate-level plausibility for functional-feed effects.

These bioactive compounds also create practical dose limits. Condensed tannins can form complexes with protein and reduce excessive ruminal protein degradation, but excess tannins may depress intake, inhibit digestive enzymes or alter mineral use. The nutritional effects of tree-leaf tannins are therefore dose-, structure- and species-dependent (24). The active compounds in paper mulberry should not be framed as uniformly beneficial; they form a functional-antinutritional continuum that must be controlled by processing and diet formulation. Quantitative reporting of paper-mulberry bioactives remains uneven. Available feed-oriented and phytochemical studies show that reported flavonoid, phenolic and tannin values vary with plant fraction, harvest stage, processing state and analytical method (7, 19–23, 25–27). Therefore, these data should be used to support dose-boundary discussion rather than pooled dose–response conclusions. A summary of available quantitative data is provided in Supplementary Table S2.

## Ensiling and fermentation as enabling technologies

Direct feeding of paper mulberry is limited by high moisture, buffering capacity, protein hydrolysis, variable palatability and storage instability. Ensiling and fermentation are therefore enabling technologies rather than ancillary processing steps. Silage additives, including lactic acid bacteria, heterofermentative lactic acid bacteria, chemical additives and enzymes, can reshape fermentation pattern, aerobic stability and feeding value (28). In paper mulberry, long-read sequencing has shown a shift from complex epiphytic communities towards lactic-acid-bacteria-dominated communities, with carbohydrate- and amino-acid-metabolism gene clusters associated with final fermentation quality (15). This moves paper mulberry silage from an empirical technology towards a microbiome-explainable process.

Additive studies further show that paper mulberry silage quality can be actively modulated. Lactic acid bacteria improve silage quality and feeding value, and different inoculants can alter pH, lactic acid, ammonia nitrogen and aerobic stability (29, 30). These findings support a simple requirement: paper mulberry silage studies should not report only ensiling duration and additive identity, but also fermentation quality.

Fermented total mixed rations containing paper mulberry are closer to commercial application. Replacing alfalfa with paper mulberry in total mixed ration silage affects ensiling characteristics, protein degradation and *in vitro* digestibility; in paper-mulberry fermented total mixed rations, the non-fibrous carbohydrate/neutral detergent fibre (NFC/NDF) ratio and compound inoculant level jointly influence pH, organic acids and mycotoxin content (10, 31). Across extracted studies (Table 3), leaf silage after 30–60 days contained approximately 21.21–25.29% crude protein and 30.43–33.11% neutral detergent fibre, whereas whole-plant silage contained 18.10–36.81% dry matter in fresh matter, 12.26–27.40% crude protein and 39.50–58.78% neutral detergent fibre. Thus, the nutritional and fermentation outcomes of paper mulberry silage remain strongly governed by plant fraction, harvest and dry-matter level (11, 29).

**Table 3:** Nutrient characteristics of paper mulberry silage in different plant parts and ensiling durations.

Reference	Plant part	Ensiling duration	DM (% FM)	% DM				
				CP	EE	NDF	ADF	Ash
Zhao et al. (76)	Leaf	30 d	—	21.21	—	31.58	14.43	13.98
Hao et al. (11)	Leaf	45 d	—	25.29	—	30.43	15.85	—
Zhao et al. (76)	Leaf	60 d	—	21.54	—	32.27	15.43	14.10
Wang et al. (69)	Leaf	60 d	—	21.63	—	33.11	24.28	—
Zhang et al. (41)	Whole plant	30 d	24.20	16.80	2.28	49.10	38.10	9.70
Dong et al. (71)	Whole plant	30 d	19.40	27.40	—	41.60	13.40	—
Dong et al. (71)	Whole plant	45 d	18.40	26.80	—	40.40	12.80	—
Si et al. (9)	Whole plant	45 d	—	15.01	—	58.78	32.15	6.17
Dong et al. (71)	Whole plant	60 d	18.10	26.20	—	39.50	12.40	—
Si et al. (72)	Whole plant	60 d	21.19	15.97	3.34	45.50	29.02	11.57
Sun et al. (29)	Whole plant	60 d	36.81	12.26	—	49.07	24.17	8.38

ADF, acid detergent fiber; CP, crude protein; DM, dry matter; EE, ether extract; FM, fresh matter; NDF, neutral detergent fiber. Fermentation-quality indicators, including pH, organic acids, ammonia nitrogen/total nitrogen, aerobic stability and mycotoxin data, were not consistently reported across the extracted studies and therefore were not tabulated as separate columns.

**Figure 1:**
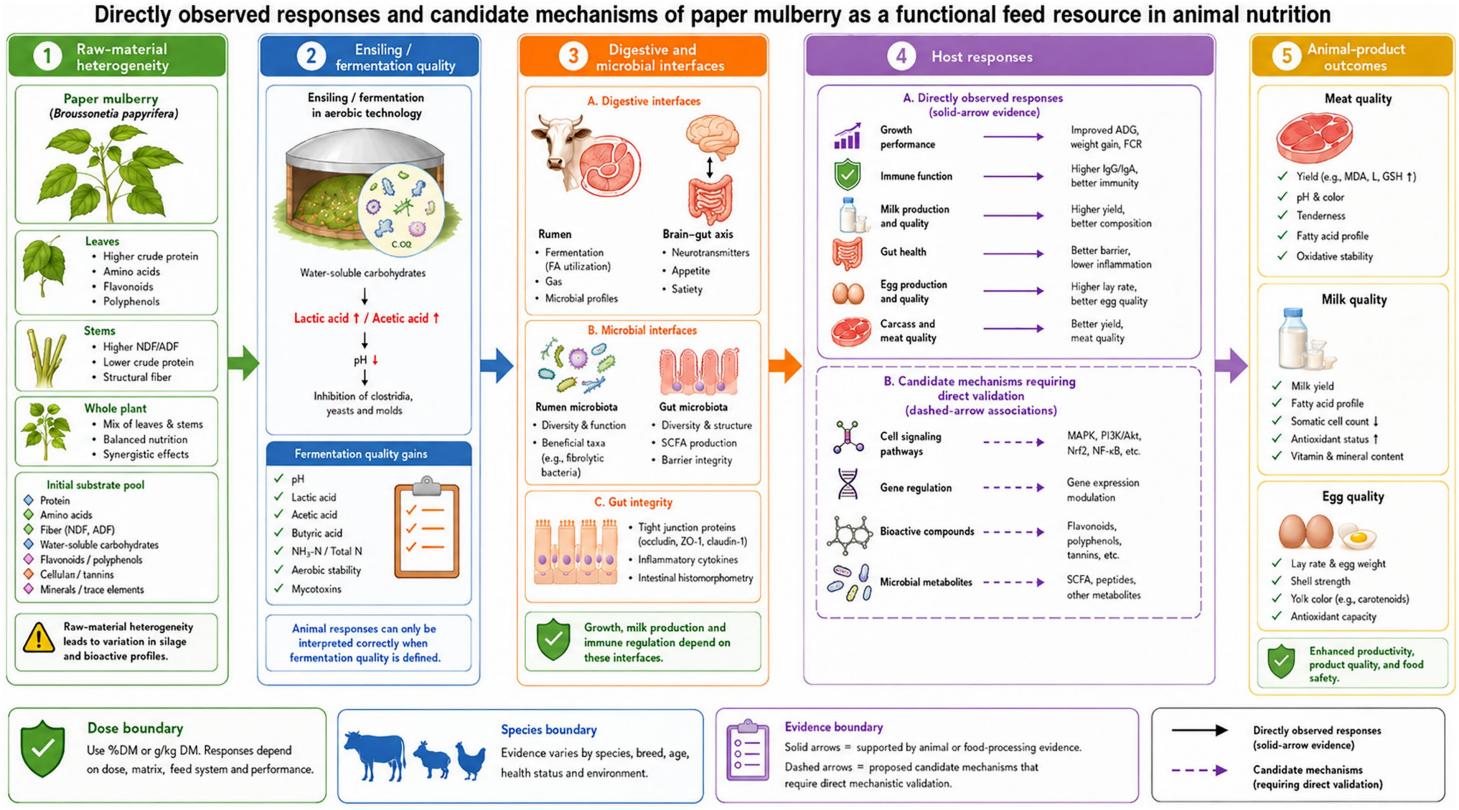
Directly observed responses and candidate mechanisms of paper mulberry as a functional feed resource in animal nutrition.

Fermentation quality also determines whether animal responses can be interpreted. If a trial reports lower intake or growth after paper mulberry feeding but does not provide pH, lactic acid, butyric acid, ammonia nitrogen/total nitrogen and mycotoxin data, it is difficult to know whether the response reflects the plant itself, processing failure or an imbalanced diet. Only when silage quality reaches a defined standard can animal responses be interpreted as genuine interactions between paper mulberry substrates and animal physiology (see Figure 1).

## Ruminants provide the strongest application evidence

Ruminants currently provide the most coherent evidence base because their rumen microbiome can ferment structural carbohydrates, while disruptions in fibre supply or fermentation balance alter rumen pH and microbial ecology (32). In this physiological context, the fibre, protein and moderate tannin fractions of paper mulberry can be used or modulated by the host-microbiome system. In goats (Table 4), replacing whole-plant maize silage with 15, 30% or 45% paper mulberry increased final body weight, average daily gain and feed intake, improved the feed conversion ratio, increased nitrogen intake, urinary nitrogen and nitrogen retention, improved crude-protein digestibility, and increased rumen pH and microbial protein (8). In dairy and beef cattle, paper mulberry silage can maintain milk composition while influencing antioxidant status, milk fatty acids, growth, meat amino acids and fatty-acid composition (9, 16).

**Table 4:** Evidence extracted from ruminant studies of paper mulberry.

Animal/Stage	Form and dose	Outcome	Reference
Dairy cows	Whole-plant silage, 5, 10, and 15% of TMR	72 Holstein cows; DMI and milk SCC decreased; milk yield, fat, protein, lactose and feed efficiency were not significantly changed; 10–15% increased serum IgA/IgG and milk PUFA; 15% increased CAT, SOD, and TAC and reduced 8-OHdG.	Si et al. (9)
Dairy cows	Leaf silage, 5, 10, and 15% of TMR	96 Holstein cows; DMI decreased but feed efficiency improved; 10 and 15% increased milk yield by 13.73 and 15.92%; serum SOD increased by 31.30 and 61.88%; MDA decreased by 36.68 and 31.88%; milk PUFA increased and SCC decreased.	Wang et al. (36)
Dairy goats	Hybrid paper mulberry fermented feed, 10% of diet	Milk yield increased; milk fat, solids, protein, lactose and amino-acid composition were not significantly changed; milk C18:3n-3 increased and SFA decreased; flavonoid extracts were linked to milk-fat synthesis genes.	Zhao et al. (37)
Goats	Whole plant, 15, 30, and 45% replacing whole-plant maize silage	40 Huanghuai white goats; FBW, ADG and ADFI increased and FCR decreased; nitrogen intake, urinary nitrogen and nitrogen retention increased; CP digestibility increased whereas DM, NDF and ADF digestibility were unchanged; rumen pH and MCP increased; NH_3_-N was unchanged; longissimus lumborum pH and redness increased and lightness decreased.	Hua et al. (8)
Goats	Whole-plant silage, 12.15% of TMR	FBW, ADG and FCR were unchanged, ADFI decreased; apparent digestibility of DM, CP, NDF, ADF and EE was unchanged, suggesting that high leaf proportion or palatability may constrain intake.	He et al. (40)
Heifers	Silage, 25, 50 and 75% replacing whole-plant maize silage	Apparent digestibility of NDF, ADF and CP increased, whereas EE digestibility was unchanged; rumen pH, acetate, propionate, isobutyrate, butyrate and valerate were broadly stable; NH3-N decreased; serum AST, ALT, urea, ALP, TP, ALB, and TC were unchanged.	Wen et al. (34)
Heifers	Silage, 25, 50 and 75% replacing whole-plant maize silage	Serum SOD, GPX, CAT and TAC were unchanged, MDA decreased; IgA, IL-1*β* and IL-4 increased, while IL-2, IL-6, and IL-17 were unchanged; faecal microbiota and hindgut parameters changed.	Tian et al. (33)
Beef cattle	Silage, 5, 10 and 15% of TMR	64 Angus cattle; 15% increased FBW, 5–15% increased ADG and DMI, and 10–15% increased FCR; SOD and TAC increased, MDA and 8-OHdG decreased; muscle lightness increased, 15% decreased pH and drip loss, 10–15% increased PUFA and 15% increased DHA.	Tao et al. (16)
Beef cattle	Silage, 5% of TMR	DMI decreased, but ADG and FCR were unchanged; longissimus dorsi pH, drip loss, cooking loss, lightness, chroma, moisture, CP, EE, ash and total amino-acid indices were unchanged, suggesting a maintenance-type low-dose use.	Yi et al. (43)
*Hu* ram lambs	Hay, 30, 60 and 100% replacing peanut vine	AWG and ADG increased, final body weight and DMI were unchanged; carcass weight and loin-eye area increased; dressing percentage was unchanged; longissimus dorsi EAA, MUFA, and PUFA increased and SFA decreased; serum IgA, IgG, IgM, and TAC increased.	Sheng et al. (38)
*Hu* sheep	Whole-plant fermented feed, 5, 10, and 15% of diet	ADG and feed efficiency increased and ADFI was unchanged; longissimus dorsi CP, TAA, EAA, flavour-related amino acids, UFA, MUFA, PUFA, and total fatty acids increased; moisture decreased; EE, ash, calcium and phosphorus were unchanged.	Su et al. (42)
*Dorper x Hu* lambs	Fermented feed, 0, 6, 18, and 100%	The 18% group had the highest beneficial fatty acids and the lowest harmful fatty acids; the 100% group had the smallest fibre diameter and area; RNA-seq of longissimus dorsi identified 443 differentially expressed genes enriched in fatty-acid metabolism, arginine/proline metabolism and PPAR signaling.	An et al. (18)

ADF, acid detergent fibre; ADFI, average daily feed intake; ADG, average daily gain; ALB, albumin; ALP, alkaline phosphatase; ALT, alanine aminotransferase; AST, aspartate aminotransferase; CAT, catalase; CP, crude protein; DHA, docosahexaenoic acid; DM, dry matter; DMI, dry-matter intake; EAA, essential amino acids; FBW, final body weight; FCR, feed conversion ratio; GSH-Px, glutathione peroxidase; 8-OHdG, 8-hydroxy-2′-deoxyguanosine; IgA, immunoglobulin A; IgG, immunoglobulin G; IL, interleukin; MCP, microbial crude protein; MDA, malondialdehyde; MUFA, monounsaturated fatty acids; NDF, neutral detergent fibre; NH_3_-N, ammonia nitrogen; PPAR, peroxisome proliferator-activated receptor; PUFA, polyunsaturated fatty acids; RNA-seq, RNA sequencing; SCC, somatic cell count; SFA, saturated fatty acids; SOD, superoxide dismutase; TAA, total amino acids; TAC, total antioxidant capacity; TC, total cholesterol; TMR, total mixed ration; TP, total protein; UFA, unsaturated fatty acids.

Heifer studies provide more direct evidence linking rumen and hindgut effects. Replacing 25, 50% or 75% of whole-plant maize silage with paper mulberry silage maintained conventional serum biochemical indices but reduced malondialdehyde (MDA), increased immunoglobulin A (IgA), interleukin-1β (IL-1β) and interleukin-4 (IL-4), and altered hindgut parameters and fecal bacterial communities (33). At the same replacement gradients, another study reported higher apparent digestibility of neutral detergent fibre, acid detergent fibre and crude protein, stable rumen pH and major volatile fatty acids, and lower ruminal ammonia nitrogen (34). Together, these data extend paper mulberry action from ruminal protein and fibre fermentation to hindgut microbiota and systemic metabolism.

In dairy animals, the main added value appears to lie in milk quality. Milk-fat synthesis depends on networks controlling fatty-acid uptake, *de novo* synthesis, desaturation, triglyceride synthesis and milk-fat-globule secretion (35). In dairy cows, paper mulberry silage studies have measured dry-matter intake, milk composition, antioxidant status and milk fatty-acid profiles. In a 96-cow Holstein study with 0, 5, 10 and 15% paper mulberry leaf silage in total mixed rations, milk yield increased by 13.73 and 15.92% in the 10 and 15% groups, respectively; serum superoxide dismutase increased by 31.30 and 61.88%, and milk polyunsaturated fatty acids also increased (36). In dairy goats, paper mulberry fermented feed and flavonoid extracts were linked to milk yield, milk fatty-acid synthesis and lipid-synthesis genes in mammary epithelial cells (37). Milk fatty-acid composition, somatic cell count, antioxidant status and mammary health may therefore be more informative endpoints than milk yield alone.

In meat ruminants, carcass responses are generally less informative than meat-quality and fatty-acid endpoints. In 64 Angus beef cattle given 0, 5, 10% or 15% paper mulberry silage in total mixed rations, the 15% group had higher final body weight, the 5–15% groups had higher average daily gain and dry-matter intake, and the 10–15% groups had higher feed conversion ratio; in meat, lightness increased, while pH and drip loss decreased in the 15% group, polyunsaturated fatty acids increased in the 10–15% groups and docosahexaenoic acid (DHA) increased in the 15% group (16). In Hu lambs, replacing peanut vine with 30, 60% or 100% paper mulberry hay increased average weight gain and average daily gain, carcass weight and loin-eye area, and increased muscle essential amino acids, monounsaturated fatty acids and polyunsaturated fatty acids while reducing saturated fatty acids (38). Additional beef and sheep studies that include histology, digestibility and intestinal-health endpoints help to interpret these meat-quality phenotypes (39, 40).

These ruminant data do not imply that paper mulberry can replace conventional roughage at high levels without constraint. Intake and production responses may increase, remain stable or decrease across studies, probably because of differences in plant fraction, silage quality, tannin content, replacement target, dietary protein and fibre balance. A more defensible conclusion is that paper mulberry has partial replacement and functional-modulation value in ruminants, but its boundary is defined by the diet system rather than by the ingredient alone.

Goat and sheep studies also show that the application value of paper mulberry is not restricted to dairy cows and beef cattle. In dairy goats, approximately 19.8% paper mulberry silage replacing alfalfa hay improved feed conversion and economic indices and enhanced antioxidant capacity (41). In Hu sheep, 5, 10 and 15% fermented paper mulberry increased average daily gain and feed efficiency without affecting feed intake; in longissimus dorsi muscle, crude protein, total amino acids, essential amino acids, flavour-related amino acids, unsaturated fatty acids, monounsaturated fatty acids, polyunsaturated fatty acids and total fatty acids increased (42). These studies broaden the evidence base for small-ruminant systems.

Recent work also places paper mulberry within more complex roughage-replacement systems. Replacing rice straw with peanut vine and paper mulberry silage reduced soybean meal use in beef-cattle diets, suggesting that paper mulberry may contribute to regional roughage and protein-feed substitution rather than replacing only one ingredient (43). Kazakh lamb studies have linked paper mulberry silage to growth, blood physiological and biochemical indices, immune response, antioxidant capacity and rumen bacteria (44). However, the dietary backgrounds and replacement logic differ across these studies and should not be merged into a single effect estimate.

## Pigs, poultry and other monogastric species

Compared with ruminants, monogastric species are more constrained by fibre, tannins and fermentation quality. Pig and poultry applications should therefore not pursue high replacement levels, but should integrate fermentation treatment, dose gradient, digestible nutrients and product quality into a single evidence chain. In finishing pigs fed low-protein diets (Table 5), 10% whole-plant fermented paper mulberry did not adversely affect average daily gain, feed intake or feed/gain ratio. It also reversed the increases in backfat thickness and fat percentage caused by low protein and increased longissimus dorsi intramuscular fat and free amino acids (45). A mechanism-oriented trial showed that 10% whole-plant fermented paper mulberry mitigated low-protein-diet-induced fat deposition, increased colonic acetate and propionate, reduced colonic lipogenic gene expression and reshaped the Bacteroidetes/Firmicutes structure (46). These results point to a gut-metabolic rather than simple growth-promoting effect.

**Table 5:** Evidence extracted from monogastric, poultry, aquaculture, and rabbit studies.

Animal/Stage	Form and dose	Outcome	Reference
Finishing pigs	Low-protein diets with 10% whole-plant powder or 10% whole-plant fermented feed, 45 d	28 crossbred pigs; growth performance was unchanged; low protein increased backfat thickness and fat percentage by 18.18 and 23.48%, and 10% powder or fermented feed restored them to normal-protein levels; fermented feed tended to reduce serum urea nitrogen (−35.63%); longissimus dorsi intramuscular fat increased by 24.53%, and histidine, arginine, glycine, taurine and β-aminoisobutyric acid increased by 45.20, 30.87, 43.00, 20.64 and 40.52%.	Song et al. (45)
Finishing pigs	Low-protein diet with 10% whole-plant fermented feed, 45 d	18 crossbred barrows; low protein increased body fat and reduced colonic acetate and propionate; 10% fermented feed mitigated these effects, reduced colonic lipogenic gene expression, increased Bacteroidetes, decreased Firmicutes and reduced cadaverine and skatole.	Duan et al. (46)
Commercial pigs	20% in early phase and 30% in late phase, 103 d	72 Xiangsha commercial pigs; overall ADG was unchanged; skin thickness increased by 19.06% in treatment I; both fermented feeds increased intramuscular fat and longissimus dorsi inosine monophosphate, and treatment I increased multiple amino acids, total amino acids, flavour amino acids and essential amino acids.	Zhang et a l. (77)
Weaned piglets	3, 6, and 9% fermented feed replacing basal diet, 25 d	216 piglets, 35 days old; 3–9% reduced diarrhoea, while ADFI, ADG and F/G were unchanged; apparent digestibility of CP, EE, calcium and phosphorus decreased; 6% gave the lowest serum TC; SOD, MDA and T-AOC were unchanged; 6% was the best overall level.	Deng et al. (47)
Guangxi Sanhuang chickens	3, 6 and 9% hybrid leaf meal, 21 d	120 one-day-old chicks; 3–6% had no adverse effects on growth, serum biochemistry or intestinal morphology; 6% increased the bursa index and increased *Lactobacillus* and *Alistipes* in the caecum; 9% increased ALT, reduced ADG and spleen index and increased F/G.	Fang et al. (48)
Late-laying hens	1.5, 3.0, and 4.5% fermented feed replacing part of maize and soybean meal, 56 d	360 Hy-Line Brown hens, 67 weeks old; ADFI, egg weight, laying rate and feed/egg ratio were unchanged, but 4.5% reduced average daily egg production; egg shape index, shell strength, albumen height, Haugh unit, yolk colour, shell thickness and yolk cholesterol were unchanged; 3.0% reduced serum TC.	Tao et al. (49)
Laying hens	1 and 5% *Lactobacillus plantarum*-fermented feed from 23 weeks of age	288 Hy-Line Brown hens; LfBP linearly increased ADFI, FCR, average egg weight and yolk colour, while decreasing shell weight and shell thickness; serum TG decreased and HDL-C increased; 1% downregulated hepatic ACC, FASN and PPARalpha and upregulated LXR; 1% was recommended as the safer level.	Niu et al. (50)
Aged laying hens	4% silage, 65 weeks of age, 56 d	240 Hy-Line Brown hens; at day 56, laying rate, egg weight, yolk colour, shell strength and shell weight increased, dirty-egg rate decreased, but Haugh unit and yolk proportion decreased; intestinal villus height, crypt depth, tight-junction genes, microbiota and 159 serum metabolites changed.	Liang et al. (17)
Geese	Leaf meal and branch-leaf meal metabolizable-energy assay	48 Magang geese, 18 weeks old; six leaf meals averaged 22.49% CP, 13.89% crude fibre and 9.72/10.23 MJ/kg AME/TME; three branch-leaf meals averaged 16.99% CP, 25.12% crude fibre and 6.87/7.35 MJ/kg AME/TME, indicating that leaf-stem ratio governs energy use.	Zuo et al. (19)
Yangzhou geese	Silage supplementation from 28 days of age, 42 d	120 male geese; ADG increased from 60.39 to 63.44 g/day (*p* = 0.056), while final body weight, ADFI, F/G and dressing percentage were unchanged; breast-muscle redness decreased from 22.36 to 19.69, crude protein increased from 79.35 to 80.42%, and 11 differential metabolites were detected.	Wang et al. (51)
Grass carp	0, 5, 10, 15, and 20% paper mulberry, 8 weeks	600 fish; 10% or higher reduced specific growth rate and 15–20% increased feed conversion ratio; 10–15% reduced muscle crude lipid and increased crude protein; muscle water loss increased in paper mulberry groups; 20% reduced TBARS from 0.45 to 0.25 mg MDA/kg; 10–20% reduced fibre diameter and increased fibre density.	Tang et al. (52)
Growing rabbits	0, 3, 6, and 9% leaf meal, 28 d	240 weaned male Ira rabbits; growth performance was unchanged; 6 and 9% reduced serum DAO, 6% increased jejunal sIgA and 3–9% altered caecal microbiota at the phylum level, suggesting a gut-health-oriented roughage role.	Han et al. (53)

ACC, acetyl-CoA carboxylase; ADG, average daily gain; ADFI, average daily feed intake; ALT, alanine aminotransferase; ALP, alkaline phosphatase; ALB, albumin; AME, apparent metabolizable energy; DAO, diamine oxidase; FASN, fatty acid synthase; FBW, final body weight; FCR, feed conversion ratio; F/G, feed/gain ratio; HDL-C, high-density lipoprotein cholesterol; IMF, intramuscular fat; PUFA, polyunsaturated fatty acids; TC, total cholesterol; TG, triglyceride; TME, true metabolizable energy; TP, total protein; LXR, liver X receptor.

Weaned pigs require a narrower interpretation. Diets containing 3, 6% or 9% fermented paper mulberry reduced diarrhoea in weaned piglets, but also reduced apparent digestibility of crude protein, ether extract, calcium and phosphorus; the 6% replacement level provided the best overall outcome (47). This result is important because a gut-health phenotype does not necessarily coincide with improved nutrient digestibility. In young animals, functional claims must therefore be evaluated alongside digestive burden.

Poultry data also show a dose boundary. In Guangxi Sanhuang chickens, 3–6% paper mulberry leaves produced no clear adverse effects on growth, serum biochemistry or intestinal morphology, whereas 9% reduced average daily gain, increased feed/gain ratio and affected liver-function-related indices (48). Higher inclusion should therefore not be assumed to deliver greater replacement value; the balance among intestinal morphology, caecal microbiota, liver function and performance is central.

Laying hens are a rapidly developing application area. In 67-week-old Hy-Line Brown hens, 1.5, 3.0 and 4.5% fermented paper mulberry replacing part of maize and soybean meal did not impair most egg-quality indicators, but 4.5% reduced average daily egg production and 3.0% reduced serum total cholesterol, making 3.0% the more suitable production level (49). A further study showed that 1% or 5% *Lactobacillus plantarum*-fermented paper mulberry increased egg weight and yolk colour and modulated serum triglyceride, high-density lipoprotein cholesterol, hepatic lipid-metabolic genes and follicular development; because the higher level may weaken shell quality, the authors recommended 1% as the safer level (50). In aged hens, 4% paper mulberry silage increased laying rate, egg weight, yolk colour and eggshell strength, reduced dirty-egg rate, and was associated with altered intestinal morphology, tight-junction genes, gut microbiota and 159 serum differential metabolites (17).

Geese, fish and rabbits help define the cross-species boundary. In geese, leaf meal had average apparent and true metabolizable energy values of 9.72 and 10.23 MJ/kg, whereas branch-and-leaf meal had values of only 6.87 and 7.35 MJ/kg, indicating that leaf-stem ratio directly affects waterfowl energy use (19). In Yangzhou geese, paper mulberry silage increased average daily gain from 60.39 to 63.44 g/day as a trend, reduced breast-muscle redness from 22.36 to 19.69, increased crude protein from 79.35 to 80.42% of dry matter and produced 11 differential metabolites (51). In grass carp, 10–20% paper mulberry reduced specific growth rate and increased feed conversion ratio, but 10–15% reduced muscle crude lipid and increased crude protein (52). In growing rabbits, 3–9% paper mulberry leaf meal did not affect growth performance, but 6–9% reduced serum diamine oxidase, 6% increased jejunal secretory IgA and all inclusion levels altered caecal microbiota (53). Cross-species use is therefore plausible, but monogastric and aquatic systems require species-specific upper limits.

## Animal-product quality as the main value-added endpoint

If paper mulberry is shown only to replace part of the roughage supply, its scientific and commercial value remains limited. The stronger value proposition lies in animal-product quality, especially the fatty-acid composition, oxidative stability, shelf life and health-related quality of meat, milk and eggs. Meat quality is shaped by muscle-fibre type, connective tissue, intramuscular fat and lipid composition; feed-derived changes can therefore act through several structural and metabolic nodes before being expressed in post-mortem quality (54). Current paper mulberry studies have measured meat colour, pH, drip loss, shear force, muscle amino acids, fatty acids, milk fatty acids, egg quality and serum lipids, but endpoint systems differ across species and remain difficult to integrate.

For meat quality, two plausible routes are antioxidant protection and lipid-metabolic regulation. Lipid oxidation impairs sensory and functional meat quality and is shaped by fatty-acid composition, oxygen, pro-oxidants and antioxidant capacity (55). Changes in meat fatty acids, drip loss or colour after paper mulberry feeding should therefore be interpreted within an oxidative-stability framework rather than as isolated traits.

Water-holding capacity and tenderness are also controlled by post-mortem energy metabolism and proteolysis. Post-mortem pH decline, myofibrillar structural change and protein degradation jointly regulate water-holding capacity, while the calpain system is central to tenderisation (56). If paper mulberry modifies pre-slaughter antioxidant status, muscle fibre structure or lipid metabolism, it could influence these post-mortem processes. Direct evidence for shelf life, thiobarbituric acid-reactive substances (TBARS), protein oxidation, myoglobin redox status and volatile flavour compounds in paper-mulberry meat studies remains limited.

Milk-quality studies have progressed beyond fat, protein and lactose towards milk fatty acids and mammary lipid synthesis. In dairy goats, paper mulberry fermented feed and flavonoid extracts were linked to genes involved in milk fatty-acid synthesis (37). Combined with work on bovine milk-fat gene networks, this suggests that future studies should test mammary epithelial fatty-acid uptake, *de novo* synthesis, desaturation and milk-fat-globule secretion rather than stopping at routine milk composition (35).

Egg quality is particularly relevant in aged laying hens, where reduced laying rate, weaker shell quality, lipid-metabolic disorder and impaired gut homeostasis frequently co-occur. Reviews of laying-hen microbiota indicate that intestinal microbes and their metabolites can influence egg quality and safety through gut-liver or gut-reproductive tract axes (57, 58). Egg-yolk lipids are dominated by triglycerides, phospholipids and cholesterol, and hepatic yolk-precursor formation changes with age (59, 60). Paper mulberry effects on egg quality should therefore be investigated along a gut microbiota-liver lipid metabolism-ovarian function-yolk quality axis rather than through egg weight, shell traits and Haugh unit alone.

Goose and fish studies further broaden product-quality endpoints. Dietary paper mulberry silage improved growth, carcass traits and meat quality in Yangzhou geese (51), and paper mulberry diets influenced growth and muscle quality in grass carp (52). Mechanistic meat-quality research is also emerging. In *Dorper x Hu* lambs fed 0, 6, 18% or 100% fermented paper mulberry, the 18% group had the highest beneficial fatty acids and the lowest harmful fatty acids, while the 100% group had the smallest muscle-fibre diameter and area; RNA sequencing (RNA-seq) identified 443 differentially expressed genes enriched in fatty-acid metabolism, arginine and proline metabolism, and peroxisome proliferator-activated receptor (PPAR) signaling (18). These results suggest that product-quality effects may arise through multiple pathways connecting fibre fermentation products, plant bioactives and host lipid metabolism.

Nevertheless, current product-quality evidence remains insufficient for strong shelf-life claims. Most animal studies measure immediate post-slaughter traits or routine egg-quality traits, with fewer data on lipid oxidation, protein oxidation, myoglobin oxidation, volatile flavour compounds, sensory evaluation or microbial spoilage during storage. Future product-quality studies should include storage loss, lipid oxidation, protein oxidation, myoglobin redox stability, volatile flavour compounds, microbial spoilage, sensory evaluation and consumer acceptance.

## Mechanistic framework: from substrate and microbiota to host cell signaling

At the feed stage, ensiling and fermentation represent the most directly documented mechanisms affecting the feeding value of paper mulberry. During ensiling, microorganisms use water-soluble carbohydrates to produce lactic and acetic acids, reduce pH and restrict undesirable microbes. Paper-mulberry silage studies show shifts from diverse epiphytic communities toward lactic-acid-bacteria-dominated communities, and additive studies demonstrate changes in pH, lactic acid, ammonia nitrogen and aerobic stability (15, 29, 30). These findings support fermentation quality as a directly documented mechanism influencing feeding value.

In ruminants, a second direct route involves rumen fermentation and nitrogen use. Moderate condensed tannins can bind feed protein at ruminal pH and may reduce excessive ruminal degradation, while tannins may also alter microbial groups involved in unsaturated fatty-acid biohydrogenation (24, 61). Paper-mulberry heifer and cattle studies reporting altered ruminal ammonia nitrogen, fibre digestibility, antioxidant status and milk or meat fatty acids are consistent with this route (16, 34).

In monogastric and hindgut-fermenting animals, hindgut and caecal responses provide another experimentally observed, although mostly associative, line of evidence. Fibre, fermentation products and plant bioactives from paper mulberry may alter microbial composition, including changes in *Bacteroidetes*, *Firmicutes*, *Lactobacillus*, *Ruminococcus* and *Blautia*, and may affect short-chain fatty acid (SCFA) production. SCFAs are key microbial metabolites linking dietary fibre to host physiology through GPR41, GPR43, GPR109A and histone-deacetylase inhibition, thereby regulating epithelial energy metabolism, tight junctions and immune responses (62). Gut microbiota, barrier-related and lipid-metabolic changes reported in pig, rabbit and laying-hen studies should therefore be interpreted through a substrate–microbiota–SCFA–barrier/metabolism axis (17, 46, 50, 53). These observations indicate microbiota-related host responses, but they do not by themselves establish directionality.

At the host signaling level, antioxidant, inflammatory and lipid-metabolic pathways provide biologically plausible explanations, but most remain candidate mechanisms in the context of paper-mulberry feeding. The Nrf2/Keap1 pathway is central to oxidative-stress regulation. After nuclear translocation, Nrf2 binds antioxidant-response elements and induces genes such as heme oxygenase-1 (HO-1), NAD(P)H quinone oxidoreductase 1 (NQO1), superoxide dismutase (SOD), catalase (CAT), glutathione peroxidase (GPX), glutamate-cysteine ligase catalytic subunit (GCLC) and glutamate-cysteine ligase modifier subunit (GCLM) (63). The *in vitro* antioxidant activity of paper mulberry flavonoids and polyphenols makes Nrf2 a plausible candidate pathway, but changes in SOD, GPX, CAT, total antioxidant capacity (T-AOC/TAC) or MDA in animal trials indicate altered systemic redox status and do not by themselves prove direct Nrf2 activation.

Inflammatory signaling provides another candidate route. NF-κB is a central transcriptional link between external stimuli and inflammatory gene expression. Toll-like receptor 4/myeloid differentiation primary response 88 (TLR4/MyD88) can activate the inhibitor of nuclear factor kappa-B kinase (IKK) complex, degrade inhibitor of kappa B (IkB) and release NF-κB p65 for nuclear translocation, inducing tumor necrosis factor-alpha (TNF-*α*), interleukin-1β (IL-1β), interleukin-6 (IL-6), cyclooxygenase-2 (COX-2) and inducible nitric oxide synthase (iNOS) (64). Paper mulberry polyphenols, flavonoids and short-chain fatty acids may affect animal health by dampening inflammatory signaling, strengthening barrier function or altering immune-cell activity, but tissue-specific validation is still needed.

Lipid-metabolic pathways may also help explain changes in fat deposition, milk fatty-acid synthesis and egg or meat lipid profiles. AMP-activated protein kinase (AMPK), peroxisome proliferator-activated receptor (PPAR), sterol regulatory element-binding protein 1 (SREBP1), fatty acid synthase (FASN), stearoyl-CoA desaturase 1 (SCD1), lipoprotein lipase (LPL) and fatty acid-binding protein (FABP) form a network linking energy sensing, fatty-acid oxidation, fatty-acid synthesis, desaturation and transport. AMPK is a nutrient and energy sensor that maintains energy homeostasis (65). Effects of paper mulberry on fat deposition in pigs, milk-fat synthesis in goats and lipid metabolism in laying hens can be placed within this network, although the dominant tissue node may differ: liver and adipose tissue in pigs, mammary epithelium in dairy goats, and the liver-ovary axis in laying hens.

Future mechanistic studies need to measure pathway activation in target tissues, including protein expression, nuclear translocation, target-gene regulation or functional inhibition. Bioactive-fraction separation, flavonoid or tannin add-back studies, microbiota-transfer experiments and coordinated transcriptomic, proteomic, lipidomic and metabolomic measurements would help separate nutritional effects from bioactive or microbiota-mediated effects.

## Practical implementation in animal production

The practical relevance of paper mulberry depends on how the resource is produced, preserved and incorporated into a complete feeding system. Commercial use requires stable biomass supply, an appropriate harvest stage, a suitable leaf-to-stem ratio, reliable preservation, manageable transport distance, realistic labour demand and economic competitiveness with conventional feed resources such as maize silage, alfalfa hay, rice straw, peanut vine or soybean meal. Because fresh paper mulberry has high moisture and limited storage stability, wilting, ensiling or fermentation will usually be required before large-scale use. Routine evaluation should include dry-matter yield, harvest frequency, fermentation success, aerobic stability, mycotoxin risk and nutrient consistency (10, 20, 28, 31).

Based on the evidence reviewed above, the approximate ranges summarized in Table 6 should be interpreted as evidence-informed boundaries rather than universal recommendations. They are intended to guide formulation decisions and identify possible safety margins, not to replace diet balancing, fermentation-quality assessment or species-specific validation.

**Table 6:** Practical interpretation of approximate paper mulberry inclusion ranges by animal group.

Animal group	Approximate range supported by current studies	Main caution
Ruminants	Often 5–15% of TMR DM or partial roughage replacement; higher replacement has been tested in specific goat/sheep systems	Depends strongly on plant fraction, silage quality, basal diet and tannin load
Finishing pigs	10% fermented whole-plant material in low-protein diets	Evidence mainly from specific diet backgrounds; digestibility and carcass fat responses need confirmation
Weaned piglets	6% performed best among 3–9% tested levels	Diarrhoea reduction may coexist with reduced nutrient digestibility
Broilers/local chickens	3–6% leaf material appeared safer than 9%	Higher inclusion may reduce growth and affect liver-related indices
Laying hens	Approximately 1–4% fermented or ensiled material depending on age and processing	Higher levels may impair laying rate or shell quality in some conditions
Fish	Use cautiously; 10–20% reduced grass-carp growth in available studies	Growth response may be negative despite changes in muscle composition

DM, dry matter; TMR, total mixed ration.

## Standardisation and future directions

Future research should move from broad claims about paper mulberry to standardised designs tailored to the specific claim being tested. For roughage replacement, studies should define the replacement target, forage-to-concentrate ratio, diet nutrient balance and fermentation quality before interpreting performance data. For functional feeding, the concentrations of tannins, total polyphenols and flavonoids should be reported together with antioxidant, immune or microbiota endpoints. For product-quality improvement, biological endpoints should be linked to shelf life, sensory attributes and commercial relevance.

Heterogeneity among studies cannot be attributed only to processing method, inclusion level or tannin concentration. Animal genotype, breed, age, physiological stage, production level, gut microbial background, basal diet composition, protein-energy balance, forage-to-concentrate ratio, housing, season, adaptation period, sample size and endpoint selection may all modify the response to paper mulberry. This is particularly important when comparing ruminants with monogastric animals, or young animals with adults.

The largest current weakness is standardisation. Studies differ in paper mulberry variety, plant fraction, harvest stage, ensiling duration, additive, replacement target and animal stage. Some report production outcomes without silage quality. Others report antioxidant or microbiota endpoints without shelf-life or sensory data. Such fragmentation weakens the scientific credibility of paper mulberry as a functional feed.

A raw-material database should include dry matter, crude protein, neutral detergent fibre, acid detergent fibre, water-soluble carbohydrates, ash, minerals, total flavonoids, total polyphenols and tannins. Mechanistic studies should further report major flavonoid, polyphenol and tannin structures. Processing studies should minimally report pH, lactic acid, acetic acid, propionic acid, butyric acid, ammonia nitrogen/total nitrogen, aerobic stability and mycotoxins. Only with these metrics can animal responses be interpreted, compared and reproduced.

Animal studies need cross-species dose–response models. In ruminants, the priority is to quantify ruminal fermentation, nitrogen balance and product fatty acids when paper mulberry replaces alfalfa, maize silage, peanut vine or rice straw. In pigs and poultry, the priority is to define non-linear relationships between fermented paper mulberry inclusion, microbiota, digestibility, liver function and product quality. In laying hens, studies should focus on aged production, gut homeostasis, hepatic lipid metabolism, follicular development and egg storage quality.

Future product-quality studies should move beyond immediate post-slaughter or routine egg-quality traits. Meat studies should measure pH decline, drip loss, shear force, muscle-fibre type, TBARS, protein carbonyls, myoglobin oxidation, lipidomics and volatile flavour compounds. Milk studies should include milk fatty acids, somatic cell count, milk antioxidant capacity and mastitis risk. Egg studies should include yolk fatty acids, yolk oxidative stability, eggshell ultrastructure and storage traits. These endpoints are necessary for paper mulberry research to move beyond feed substitution into product-quality science.

The most valuable next step is not another set of isolated feeding trials, but studies that close the causal chain. Such studies should control raw material, fermentation quality, diet balance, animal microbiota, host tissue pathways and product shelf life in the same design, or use bioactive-fraction removal and add-back experiments to test the contributions of flavonoids, tannins or fermentation metabolites. Only then can paper mulberry move from regional unconventional feed to a mechanism-defined functional feed resource.

## Evidence strength and unresolved questions

Paper mulberry research must acknowledge the hierarchy of evidence. *In vivo* animal trials directly support conclusions about production performance, health and product quality. Production-setting trials reflect practical diet conditions and transferability. Phytochemistry, *in vitro* antioxidant work and canonical mechanistic studies support biological plausibility, but cannot replace animal evidence. Reviews and methods papers are useful for framing concepts, but should not be used as proof of specific production effects. Direct extrapolation from *in vitro* activity to animal-product quality risks mechanistic overinterpretation.

This hierarchy determines claim strength. Stronger language is justified for effects supported by animal trials, such as altered milk fatty acids, rumen and hindgut microbiota in Holstein heifers, meat quality and gut microbiota in finishing pigs, and egg quality and caecal microbiota in laying hens (9, 17, 50). In contrast, Nrf2, NF-κB, AMPK and PPAR should be described as candidate or consistent pathways unless future paper mulberry trials directly test protein expression, nuclear translocation, target genes and functional blockade.

One unresolved issue is the classification of paper mulberry as a protein-rich roughage. Leaves and young shoots are protein-rich, but high crude protein does not equal high utilisable protein. In monogastric species, fibre and antinutritional factors limit energy and amino-acid use. In ruminants, protein value further depends on ruminal degradability, microbial protein synthesis and rumen-undegraded protein flow. Future studies should therefore report conventional protein, protein fractions, ruminal degradation, faecal and urinary nitrogen and product nitrogen deposition, rather than using crude protein alone as proof of protein-feed value.

A second issue is the directionality of bioactive compounds. Flavonoids and polyphenols are often framed as antioxidant and anti-inflammatory molecules, but their bioavailability, metabolic transformation and tissue distribution in animals are not always clear. Tannins are explicitly dual-function compounds: moderate levels may protect protein and alter fatty-acid biohydrogenation, whereas high levels may depress intake and digestibility. Without measurements of total tannins, condensed tannins, flavonoids and polyphenols, animal responses cannot be assigned to nutrition, fermentation or bioactive chemistry.

A third issue is the commercial meaning of product-quality endpoints. Many studies report statistical significance, but it is often unclear whether the effect size is perceptible to consumers, processors or nutrition-label claims. Changes in fatty-acid proportions, meat colour, drip loss or yolk lipid profiles need to be connected to shelf life, sensory traits and industrial usability. Statistical significance should therefore be interpreted together with effect size, storage performance, sensory data and commercial relevance.

The overall assessment is cautiously positive. Paper mulberry has a strong resource base and a growing animal evidence base, but the field has not yet reached a stage where mechanisms are fully resolved, quality standards are unified or dose boundaries are clear across species. Key questions remain: how variable are raw materials, how controllable is silage quality, how reproducible are animal responses, which mechanisms are causal and which product-quality changes are commercially meaningful. The way forward is not to overstate the value of paper mulberry, but to close the evidence chain with standardised metrics, representative studies and explicit boundaries.

## Conclusion

Paper mulberry has genuine potential as a functional woody feed resource, but its value depends on how it is cultivated, harvested, processed and included in the diet. The strongest evidence comes from ruminant studies, where paper mulberry has been associated with partial roughage replacement, maintained production, altered rumen or hindgut ecology, improved antioxidant indices and changes in meat or milk traits under specific dietary conditions. Evidence in pigs, poultry, laying hens, waterfowl and fish is useful but more constrained by fermentation quality, fibre load, dose and species-specific digestive capacity.

Future studies need sharper designs: which plant fraction, processed by which method, fed to which animal at which dose, through which mechanism, improves which product-quality trait. With raw-material databases, silage-quality grading, cross-species dose–response studies, product-quality endpoints and multi-omics causal validation, paper mulberry could become a platform connecting sustainable feed resources, animal health and high-quality animal-product production.
