# Supplementary material for: Paper mulberry in animal nutrition: processing, host responses, and product quality
Source: Front Vet Sci. 2026 Jul 29;13:1907163. doi: 10.3389/fvets.2026.1907163 (PMC13461557; doi:10.3389/fvets.2026.1907163)
Supplement: Supplementary file 1 [file Table_1.DOCX]

**Supplementary Figure S1. Literature selection flow diagram**

Evidence allocation for this structured narrative review of paper mulberry in animal nutrition

**Supplementary Materials**

**Supplementary Table S1. Search strategy, selection logic and evidence-appraisal framework for this structured narrative review.**

| Step | Operational definition |
| --- | --- |
| Review type | Structured narrative review; not a PRISMA meta-analysis and not a pooled quantitative synthesis. |
| Databases | PubMed, Web of Science, Google Scholar, CrossRef and China National Knowledge Infrastructure (CNKI). |
| Search period | Core paper-mulberry animal-production and processing literature: 1 January 2010 to 1 June 2026. Earlier records were retained only for foundational biology, tannin nutrition, rumen ecology, product-quality mechanisms or cell-signaling background. |
| Search string | ("*Broussonetia papyrifera*" or "paper mulberry") and ("animal nutrition" or feed or silage or fermentation or ruminant or cattle or dairy cow or goat or sheep or pig or poultry or broiler or laying hen or goose or rabbit or fish or aquaculture) and ("growth performance" or digestibility or "gut microbiota" or antioxidant or inflammation or "meat quality" or "milk quality" or "egg quality" or "fatty acid" or "shelf life"). |
| Language restriction | English and Chinese publications were considered because bibliographic information, full texts and methodological details could be reliably verified in these languages. |
| Inclusion criteria | Studies were retained when paper mulberry was evaluated as a feed ingredient, forage, silage, fermented feed, total mixed ration component, animal-production input or biologically active feed material with extractable information on species, form, dose/replacement level, processing method and at least one production, health, microbiota, fermentation or product-quality endpoint. |
| Exclusion criteria | Human medicine, pure plant physiology, unrelated food chemistry, in vitro antioxidant assays without animal-feeding relevance, duplicate records and studies without extractable animal-production or feed-processing information. |
| Cited literature | The cited literature included direct animal-feeding/application studies, feed-resource and processing studies, and background mechanistic references. Earlier foundational records were retained only when needed for tannin nutrition, rumen ecology, product-quality biology or canonical signaling interpretation. |
| Evidence hierarchy | Level 1: in vivo animal-feeding studies. Level 2: feed-resource, ensiling and fermentation studies. Level 3: product-quality and host-physiology studies. Level 4: phytochemistry, in vitro antioxidant and canonical signaling studies. |

**Supplementary Table S2. Quantitative reporting of flavonoids, phenolics/polyphenols and tannins in paper mulberry materials.**

| Material / plant part | Harvest or processing state | Total flavonoids / flavones | Total phenolics / polyphenols | Tannins / condensed tannins | Interpretation and limitations | Reference |
| --- | --- | --- | --- | --- | --- | --- |
| Leaves; leaf meal from six origins | Dried leaf powder; origin varied (Hunan, Hubei, Henan, Hebei, Anhui and Sichuan) | 327.47-890.29 microgram/g; mean 541.89 microgram/g total flavones | NR | 227.72-238.86 microgram/g; mean 233.07 microgram/g tannins | Plant part is explicit. Useful for comparing leaf meal with branch-leaf meal, but values should not be pooled with mg/g or % DM assays because extraction and calibration differ. | [1] |
| Twigs plus leaves; branch-leaf meal from three origins | Dried branch-leaf powder; Guangdong samples | 560.10-806.74 microgram/g; mean 652.89 microgram/g total flavones | NR | 210.32-223.49 microgram/g in quantified samples; mean 216.91 microgram/g tannins | Branch-leaf material had higher mean total flavones but lower energy value than leaf meal, showing that bioactive concentration does not override fibre-driven feed value. | [1] |
| Whole harvested hybrid paper mulberry | Fresh material cut at 20, 35 and 50 cm stubble heights | NR | NR | 0.87, 0.98 and 1.08% DM total tannins at 20, 35 and 50 cm, respectively | Harvest height changed total tannins together with crude protein and fibre; the unit is % DM and is not directly comparable with microgram/g extract-based values. | [2] |
| Whole harvested hybrid paper mulberry | Ensiled for 45 d after cutting at 20, 35 and 50 cm stubble heights | NR | NR | 1.38, 1.60 and 1.94% DM total tannins at 20, 35 and 50 cm, respectively | Ensiling did not remove the need to quantify tannin load; in this study, reported total tannin values were higher after ensiling than in fresh material. | [2] |
| Paper mulberry leaves | Dried leaf material in a comparative woody-feed evaluation | 21.01 +/- 1.63 mg/g flavones | 10.66 +/- 0.07 mg/g total phenols | 8.12 +/- 0.57 mg/g tannins | This source jointly reports flavones, total phenols and tannins for leaves, but because it is thesis-level evidence, it should be treated as supportive and placed in supplementary material rather than used as a primary basis for feeding recommendations. | [3] |
| Paper mulberry leaves | Leaf material summarized from HPLC-based literature | 15.31 g/kg total flavonoids; major reported components included quercetin, isoliquiritigenin and daucosterol | NR | NR | Secondary evidence only; should not be used for dose-boundary inference unless the original HPLC source is verified and added to the reference list. | [4] |
| Paper mulberry material with plant fraction not fully resolved in the extracted text | Reported in a feed-resource context; plant fraction and processing basis should be checked before use | NR | NR | Approx. 15.5 mg/g tannins reported in a thesis literature summary; condensed tannins approx. 6.96-7.09% reported from Obour et al. as cited there | Do not use as a part-specific dose boundary unless the original material description is verified. It can be cited only as a caution that tannin values vary widely with material definition and method. | [5, 6] |
| Leaves, roots, fruits, bark or other medicinal extracts | Solvent extracts or isolated compounds rather than feed materials | Individual flavonoids/phenolics reported; total feed-basis concentration often NR | Individual phenolic compounds reported; total feed-basis concentration often NR | NR | Use for substrate-level plausibility and chemical diversity, not for animal dietary dose boundaries unless dry-matter feed concentration is reported. | [7-9] |

NR, not reported. Values are presented in their original reported units and should not be pooled unless sample basis, extraction procedure and calibration standards are harmonised. DM, dry matter; HPLC, high-performance liquid chromatography.

References

1.	Zuo, X., et al., *Determination of nutritional components and goose metabolizable energy of paper mulberry leaf meal and branch-and-leaf meal from different origins.* Chinese Journal of Animal Nutrition, 2018. **30**(7): p. 2823-2830.

2.	Hao, Y., et al., *Effects of different stubble heights on yield, nutrient composition, silage quality and in vitro fermentation of paper mulberry.* Chinese Journal of Animal Nutrition, 2020. **32**(5): p. 2387-2396.

3.	Jiang, Z., *Evaluation of the nutritional value of forage mulberry, paper mulberry and elm leaves and their feeding effects in rabbits*. Jiangsu University of Science and Technology.

4.	Zhang, H., et al., *Nutritional value of paper mulberry leaves and their application in animal production: A review.* Chinese Journal of Animal Nutrition, 2020. **32**(9): p. 4086-4092.

5.	Wang, H., *Effects of lactic acid bacteria isolated from paper mulberry on the silage quality of paper mulberry leaves*. 2022, Anhui Agricultural University.

6.	Obour, R., S. Oppong, and I. Abebrese, *Chemical composition and nutritive value of an invasive exotic species Broussonetia papyrifera in Ghana.* Journal of Natural Sciences Research, 2017. **7**(20): p. 45-51.

7.	Wang, G.W., B.K. Huang, and L.P. Qin, *The genus Broussonetia: a review of its phytochemistry and pharmacology.* Phytotherapy Research, 2012. **26**(1): p. 1-10.

8.	Yang, C., et al., *Isolation and Characterization of New Phenolic Compounds with Estrogen Biosynthesis-Inhibiting and Antioxidation Activities from Broussonetia papyrifera Leaves.* PLOS ONE, 2014. **9**(4): p. e94198.

9.	Cao, X., et al., *Antioxidant evaluation-guided chemical profiling and structure-activity analysis of leaf extracts from five trees in Broussonetia and Morus (Moraceae).* Scientific reports, 2020. **10**(1): p. 4808.
